# Legal advice and care-effective use of care and case management: limits, risks and need for change (funded by the innovation Fund of the Federal Joint Committee (G-BA) according to §§ 92a and 92b of the fifth book of the German social code (SGB V). Funding code 01NVF17029 (RubiN) regional uninterrupted in the network)

**DOI:** 10.1186/s12913-022-08844-z

**Published:** 2022-11-29

**Authors:** Thomas Ruppel, Max Georg Hügel, Simone Gloystein, Neeltje van den Berg

**Affiliations:** 1 University of Greifswald, Greifswald, Germany; 2 Bucerius Law School, Hamburg, Germany

**Keywords:** Care and case management, Legal requirements, Social law, Public health, Geriatrics

## Abstract

**Introduction:**

An important dimension of care and case managers is to support geriatric patients in obtaining social services in medical, nursing, therapeutic and social fields. To this, they advise and represent their patients.

**Methods:**

The documentation of patient contacts with case managers of a network of physicians was evaluated. In particular, activities involving legal advice were analysed in detail, compared with the current legal situation in Germany and evaluated. In addition, qualitative expert interviews were conducted. The content and the legal requirements of legal services law were determined by applying legal interpretation methods (esp. wording, telos, systematics). The results of the evaluation of the documentation were compared with legal requirements.

**Results:**

Care and case management touches activities in some fields of action without having a legal basis in legal services law. This leads to the fact that these services may not be provided and to - uninsured and uninsurable - liability risks.

**Discussion:**

With the introduction of care and case management into standard care, both social law and the Legal Services Act must be adapted to enable the legally compliant use of care and case managers. Otherwise, certain services that are useful for the care of patients may not be provided.

## Introduction

The project was carried out in Germany, so some aspects, such as occupational profiles and legal frameworks, have a German context. Care and case management organises and coordinates the care of patients with complex treatment needs and establishes treatment pathways for patients. Care and case management is often used for geriatric patients. They support patients with different care needs and in various living situations to find their way through the jungle of welfare state services.

For the implementation of the intervention in the following project, health professionals were qualified according to a modular curriculum that was used for the training of case managers. Content was taught on various fields of action, such as health and project management, risk identification, creation of clinical pathways, case management or risk management. The qualification program for the prospective case managers consisted of an online part (40 hours) and a practical training in presence (40 hours). The online training consisted of independent completion of a homework assignment, video lessons, and video tutorials. Participants completed the hands-on training in the form of a one-week classroom workshop. The two training formats were followed by the “extended training in practical everyday life”. The case managers brought relevant experience from their initial training as nurses, therapists or physician assistants. They provided consultation and coordination services on medical, therapeutic, nursing and social aspects across occupational groups, sectors and social codes.

Management of the often complex care of geriatric patients by care and case managers or other coordinating functions has often positive effects on both the health situation of patients and on the [1] burden [2] of relatives [3]. Through care and case management, tasks in the health care system are better assigned to the right places and at the right times, which for example, also leads to a reduction in the workload of general practitioners [4, 5].

In many cases, it makes organisational sense for care and case managers to work in networks of doctors rather than for individual practices. The care of geriatric patients in physician or care networks improves, for example, the utilization of services for patients with dementia [6, 7].

Since care and case managers provide advice at the interfaces of medical, social and legal issues, this raises the question of the legal limits of legal advice by care and case managers and a possible need for legal adaptation, since otherwise effective use of care in standard care cannot be guaranteed and would be associated with liability risks for case and care managers.

In this paper, those selected activities of case managers in the RubiN project (regional network of care, funded by the Innovation Fund of the G-BA) that are at the interface of legal advice and representation, are analysed in terms of content and compared with the current legal situation in Germany.

## Methods

The project “RubiN -continuous care in regional networks” is designed as a prospective controlled intervention study to investigate whether multi-professional, cross-sectoral and assessment-based car and case management leads to an improvement in the identification, care situation and health status of geriatric patients who still living in their own homes in different regions in Germany. In the RubiN project, geriatric patients of the intervention group have been cared for by care and case managers over a period of twelve months. On the basis of an extensive geriatric assessment, gaps in care and individual patient needs and resources were identified. On the basis of the assessment results, individual treatment plans were drawn up for patients in collaboration with treating general practitioners. The effects of intervention will be evaluated in a controlled design with five intervention and three control regions. The design and methods of the Rubin project have been published [8].

For the analysis of the activities of care and case managers in relation to the legal situation, the documentation of patient contacts with case managers for a network (member of the intervention group) of the RubiN project was evaluated. In particular, the activities related to legal advice were analysed in detail, compared with the current legal situation in Germany and evaluated.

In addition to the evaluations of performance, expert interviews were conducted as open, semi-structured surveys with the project managers of intervention networks. The results of the performance evaluations were deepened and explained with these interviews.

The content and legal requirements of the Legal Services Act were determined by applying the legal methods of interpretation. Interpretation methods are legal methods used in science and legal practice to interpret the contents of norms, to exegete a text. The aim of interpretation is to determine the content, abstract concepts are thereby given a concrete meaning; they include, among others, the methods for interpreting the wording of a norm, for understanding the previous and current norm maker, for the systematic position of a provision in the structure of one and further laws, for achieving the highest possible effectiveness of constitutional requirements in lower-ranking legal provisions, etc. These methods are applied in whole or in part in the interpretation of a norm. The [9] results of the documentation evaluations and the expert interviews were compared with legal requirements [10]. Legal limits found in this way were interpreted and conclusions were drawn in terms of utilities and jurisprudence.

## Results

### Activities involving legal advice, using the example of RubiN

Data from the five physician intervention networks were evaluated. In the networks, a total of 3418 patients were recruited for the RubiN- project. After checking the inclusion criteria (age ≥ 70, score from the geriatric screening (ANGELINA) ≥ 2 from at least two different subject complexes and membership of a statutory health insurance) and taking into account revocations granted or other reasons for inactivation (before the start of intervention on the part of the patients), the data of 3059 patients were taken into account in this work. These 3059 patients (1970 were women) were followed up as part of the RubiN project. The patients were on average 81,9 years old. For the 3059 patients, a total of 34,940 contact logs with service documentation are available for the intervention period of 12 months. On average, 11,4 performance records were completed per patient during that time. The minimum number per patient is 1 and the maximum number per patient is 105 service records; 6 service records were completed most frequently. A total of 63,902 consultation contents (Table 1) were documented, of which 3170 consultation contents (Tables 2 and 3) were relevant to a possible violation of the Legal Services Act.

**Table 1:** Consultation content: (*N* = 63,902 with 23,518 contacts)

Variable	Number
Medical:	16,867
Nursing:	13,258
Therapeutic:	13,613
Social:	20,164

**Table 2:** Legal advice content with reference to health insurance (*N* = 1106 from total 24,028)

Participation in applications legal area SGB V	Number
Prescription of physical therapy	232
Prescription physiotherapy	31
Prescription of ergotherapy	37
Speech therapy	12
Aid	175
Rehab sport (partly SGB XI)	46
Hospitalization	64
Geriatric day clinic	67
Specialised geriatric rehabilitation	73
Application for treatment in a geriatric clinic	16
Outpatient nursing service - Medical treatment care	146
Outpatient care service - Domestic work	36
Household help	27
Travel costs	42
Copayment exemption	102

**Table 3:** Legal advice content with reference to long-term care insurance(*N* = 2064 from total 24,028)

Participation in applications legal area SGB XI	Number
Daycare	117
Short-term care	78
Preventive care	77
Full inpatient care	50
Home nursing	11
Care level	1107
Outpatient nursing service - Basic care	93
Low-threshold care and relief service	251
Home emergency call	130
Measures to improve the living environment	150

Table 1 first shows the total number of advisory services.

Table 2 contains that part of the counselling services that is directly relevant to the topic of legal counselling, namely the involvement of care and case managers in filing of applications, categorised according to the Social Codes V (statutory health insurance) and IX (rehabilitation and participation of people with disabilities).

Some of the services were documented as “applications”. The term “application” represents very heterogeneous activities: the search for forms, assistance in filling out the forms by care and case managers, and the application process by care and case managers as representatives of the patients. The involvement in applications in this sense covered a wide variety of service areas. The most common services were “Application for a care degree”, “Application for a prescription for physiotherapy” and “Application for aids”.

Care and case managers were also involved in 89 applications for the determination of severely disabled status and the granting of benefits under the State Law on the Blind and in one appeal procedure. Apart from social law, care and case managers also supported their patients in the drafting of living wills (137 cases), health care powers of attorney (137 cases) and guardianship orders (61 cases).

### Results of the expert interviews

An expert interview is a qualitative research method in which experts in their field are interviewed using guidelines. It is not about the person themselves, but about their expertise or their way of seeing or acting (insider knowledge) in their field of expertise. For the interviews, topics were collected beforehand, which were then discussed in a flexible framework. This was a semi-structured approach. Five expert interviews were conducted with the project managers of the practice networks participating as consortium partners in RubiN. In the course of these expert interviews, one practice network said clearly that, from the point of view of the practice network and care and case managers there, no legal advice was given; patients were only asked whether living wills and health care proxies existed, where they were stored and whether the family doctor was informed. If no living wills and health care proxies existed, they were advised to do so and provided with information material from the Federal Ministry of Justice. In response to an explicit enquiry, this network of practices stated that, in particular, no advance directives and living wills to tick off had been distributed, nor had such prefabricated precautionary documents been jointly completed. The staff had been trained by a local authority, and great care had been taken to ensure that the care and case managers knew their limits.

The managing director of another practice network stated that the care and case managers would provide advice on all relevant social security codes, in particular on basic security, help with care and the scope of the family insurance fund, but also on matters of tenancy law and housing benefit, and would provide support with applications.

In a third practice network, the interviewee stated in the interview that in the case of living wills (usually a written declaration of intent of a person in the event that he or she is no longer able to express his or her will to doctors or nursing staff) and health care proxies (an instrument of legal precaution which stipulates that the proxy handles financial, organisational and medical procedures on behalf of the person who has granted the power of attorney), reference would be made to the care associations. However, the forms of the Federal Ministry of Justice are also available. If there are problems with filling out the forms, care and case managers also provide support.

One practice network reported in the interviews that there had been requests to help with the drafting of wills, this had been refused.

### Concept of legal advice

The described counselling and representation activities of the care and case managers are legal services in the sense of § 2 para. 1 of the Legal Services Act (RDG; this regulates who and under which circumstances may provide legal services in Germany) and thus subject to registration according to §§ 3, 15b RDG:

Legal service is “any activity in concrete third-party matters as soon as it requires a legal examination of the individual case” (Section 2 (1) RDG). The requirements for the existence of a “legal examination” are quite low, which is already evident from the legislative history, in which alternative proposals such as “extensive legal examination” or “in-depth legal examination” were rejected in favour of the general, simple “legal examination”, which does not require any special characteristic [11]. Legal advice is given when the answer to a legal question is not completely clear, but can only be obtained through reflection and evaluation.

The “necessity” of a legal examination required by law contains an objective and a subjective component. The subjective component is linked to the expectations of the person seeking legal advice. If he asks specific legal questions, his expectation of receiving a legal service becomes clear. If this is the case, it is a legal service. Objective aspects such as the perception of the general public or the protective purpose of the law (what is the purpose of the law, what exactly is this law supposed to “protect”) are then no longer relevant [12].

The objective component catches in particular the cases in which the person seeking legal advice does not even know that he is asking for legal advice or even expressly wants to do without it. It consists of the values of the usual views of business dealings and the protective purpose of the RDG and is therefore ultimately paramount [13]. In this context, in particular, the express waiver of the protection of the Legal Services Act by the person seeking legal advice is not possible (cf. § 3 RDG).

As a result, it is evident that much of the work of care and case managers is likely to fall under the heading of ‘legal services’. This is indeed supported by the empirical research on which this article is based, as explained in more detail in the following section.

### Advice and representation in applications for social benefits

The evaluation of data and of interviews show that care and case managers are involved in a variety of very heterogeneous ways in making applications to different service providers. Applying the definition of legal counselling that has been elaborated, these services, both advisory and external representation, are inadmissible for lack of authorisation. This is because, according to the interviews, care and case managers act as advisors in social law matters, among other things, and support patients in filling out application forms. Such advice and support necessarily presupposes that care and case managers relate the actual circumstances of their patients to the relevant legal bases. A completely schematic application, identical for each individual case, is far from the case in this individual counselling of each patient. Moreover, since patients wanted to have concrete legal questions answered in this counselling and since, in any case, legal transactions regard professional advice in social, tenancy and other fields of law as legal advice (cf. § 11 para. 2 RDG), care and case managers provide a legal service in the sense of § 2 para. 1 RDG in such cases without having the necessary permission in the sense of § 3 RDG. From the activities actually carried out, it can be deduced that expertise was lacking, especially where legal deadlines and formal requirements had to be complied with or where knowledge of special regulations (such as in the case of fictitious authorisations) and court rulings (such as the case law of the supreme court [BGH] on requirements for living wills) was important.

As a result, the payers of the services applied for directly by care and case managers are initially obliged under current law to reject care and case managers - or more precisely: their employer - as representatives of the insured patients, since the service is provided in contravention of Section 3 of the RDG, Section 13 (5) SGB X. Procedural acts performed after rejection are invalid, but acts performed up to that point remain valid (reverse conclusion from § 13, Subsection 7, Sentence 2, SGB X) [14]. However, it is obvious that the cost units do not even notice this because the representation is not made clear to the outside; especially if, after legal advice by care and case managers, the insured patient signs the application himself. Additional risks of this legal advice are manifold, for example with regard to questions of form and time limits when filing objections or the ignorance of the fictitious approval according to § 13 Para. 3a SGB V, which is especially important in psychotherapy. In the case of this fictitious approval, if the health insurance fund does not decide on an application for benefits within a certain period of time, it is pretended that approval has been granted, the approval is fictitious (faked). In this way, there is a risk that patients’ claims will be lost or that other legal disadvantages will be caused.

### No suitable exemption rules

Apart from lawyers, legal services may also be provided, among others, by way of exception by publicly funded consumer associations, associations of voluntary welfare work pursuant to § 5 SGB XII and recognised associations for the promotion of the interests of disabled persons pursuant to § 15 (3) BGG within the scope of their tasks and responsibilities (§ 8 (1) no. 5 RDG). However, these and other exemption provisions - i.e. special permits for legal advice by non-lawyers - are not fulfilled in the case of care and case management. In particular, this service is not provided by recognised associations of independent welfare work within the meaning of SGB XII or by associations for the promotion of the interests of persons with disabilities, but is rather closely related to health services provided by health insurance. It is also not a privileged pension consultation in the sense of § 10, Subsection 1, Sentence 1, No. 2, RDG. This exceptional circumstance always requires a concrete pension reference for consultation to the social security right [15]. When creating the RDG, the legislature deliberately decided against the creation of a general social law advisor and deliberately wanted to limit the exception to pension insurance [16]. The exception regulation in § 1908f exp. 4 BGB with the consultation to precautionary powers of attorney applies only to these and only to recognized care associations. The RDG thus applies to all other providers of these services [17].

It remains questionable whether the activity according to § 5 RDG is permitted as an ancillary service to another profession or activity. According to § 5 para. 1 sentence 2 RDG, this is to be assessed “according to its content, scope and factual connection with the main activity, taking into account the legal knowledge required for the main activity”. This is because many professional activities also require the provision of peripheral legal services in order to be able to perform it in accordance with the requirements of the activity. Whether legal advice is necessary as an ancillary service to the job description results in particular from legally regulated job descriptions and job descriptions [18]. The decisive factor here is that the legal service is related to a (main) service and that it is only an ancillary service in relation to this [19].

The performance records show that counselling services take up a large share of the activities of care and case managers. Non-counselling services only accounted for a small proportion, such as referrals to network partners (*N* = 1355). Delegation services according to Annex 24 of the Bundesmantelvertrag (agreement between the Kassenärztliche Bundesvereinigung (Federal Association of Statutory Health Insurance Physicians) and the Spitzenverband Bund der Krankenkassen (National Association of Statutory Health Insurance Funds) to ensure the provision of medical care for insured persons) as physical, delegated medical services took place in the entire project in only one intervention region (*N* = 3756); here, the focus was on blood glucose measurements, blood sampling and medication checks.

The evaluation of data does not show whether possible legal counselling activities fall under “social” or “medical”, if there was a reference to benefit applications vis-à-vis health insurance funds. In the one (out of five) intervention region in which delegation services were provided, 5198 consultations (4515 consultations with patients directly, 556 consultations with relatives (relatives only), 106 joint consultations (patient and relative) and 21 without related information) were recorded with a total of 11,866 consultation contents (medical: 4803; nursing: 1039; therapeutic: 1191; social: 4833) were compared with a total of 3756 medical delegation services. Even in the intervention region where care and case managers also provided delegation services at the same time, counselling services still accounted for the majority of services; where no delegation services were provided, this is the case anyway.

## Discussion and conclusion

Care and case managers also provide legal services in their holistic counselling and assistance to patients. There is currently no legal basis for these. However, low-threshold access to counselling on social benefit entitlements and living wills and advance directives makes sense for the effective use of care and case management and the achievement of the associated goals. Otherwise, counselling would not be provided at all or would be provided by persons who are legally ignorant or not authorised to do so (relatives, doctors, care services, care support centres). A lawyer’s monopoly for counselling on this scale would presumably lead to the loss of this instrument without replacement rather than to a shift of legal counselling to the legal profession, which in itself is called upon to do this, but which is less easily accessible, especially for geriatric patients.

Counselling is the main service provided by care and case managers. The classification of legal counselling as an ancillary service could only be considered if one were to differentiate between counselling and legal counselling for care and case managers. This cannot be delineated from the service records; moreover, this differentiation would artificially split the holistic counselling approach of care and case managers. This is all the more true as the medical and social counselling tasks are inextricably linked to counselling on legal claims precisely to enforce the medical and social goals of the patients. The purpose of the RDG, which is to protect against improper legal advice by those not sufficiently qualified for this and those with liability insurance, also argues, in view of the considerable risks described, that the assumption of a legal advisory ancillary service within the meaning of § 5 RDG in addition to counselling as the main service is ruled out. Whenever legal issues are also the subject of counselling by care and case managers, the counselling activity as a whole is subject to authorisation. This leads to a considerable role conflict of care and case managers between their mediator position on the one hand and the role of the “patients advocate” on the other.

The concept of GeriNurses on which the RubiN project is based describes care and case managers as “advocates” and “representatives” of the patient and includes among their tasks (literally) the “independent application in the form of counselling and accompaniment of the Social Code”. According to this, care and case managers should also be so-called gatekeepers and, in particular, check patients’ entitlements in the health system. From the perspective of care science, care and case managers are thus given a double role; they are supposed to be both the patient’s advocate and a neutral mediator between the patient and the service providers, especially the health and long-term care insurance funds.

As comprehensible and sensible as this is from a holistic care science perspective, it meets with considerable reservations from a legal perspective. Legally, a partisan lawyer role and a neutral mediator role are categorically mutually exclusive. This is explicitly regulated, for example, in the Mediation Act (§§ 1 para. 2, 2 para. 3, 3 para. 2 MediationsG), which states that anyone who is or was already acting for a party in the same matter may not act as a neutral and all-partial mediator (extrajudicial mediator between parties). Anyone who is partisan can no longer be all-partisan or impartial.

Further problems of this double role arise from the fact that the function of care and case managers becomes contourless in the application process. If they, as mediators between the patient and the service provider, are to realise both interests equally, they would logically also have to be part of the communication process of both sides. In this case, however, an application for benefits from the patient would already be considered to have been received by the service provider when it is received by the care and case manager (§ 16 para. 2 sentence 2 SGB I); conversely, the care and case manager as the patient’s representative would also be authorised to receive authorisations and other declarations with effect for the patient - until a rejection according to § 13 para. 5 SGB X. The care and case managers would thus be responsible both to the service providers and to the patients for any delays in passing on information.

This responsibility, with the risk of considerable loss of trust and data protection problems on both sides, is problematic. Because with such an involvement of care and case managers in the treatment process, patients can never be sure whose interests the care and case managers are supposed to look after and actually do look after.

Therefore, in order to close this gap in the need for counselling, it should be considered - under the condition of sufficient qualification - to allow appropriate legal counselling services and representation in individual cases by care and case managers for their patients. The curriculum of the additional legal qualification must therefore be quite demanding and comprehensive, at least for living wills and advance directives (cf. § 1908f paras. 1 and 4 BGB), in order to do justice to the complexity of these fields of counselling. Without such a qualification, care and case managers cannot fulfil the trust placed in them by patients and the situational need for counselling. The legal power of representation should be limited to the initial procedure (application) and not also cover the appeal procedure.

One way to achieve such a design would be to supplement the opening clauses for objectively permitted legal advice in section 10, paragraph 1, sentence 1 RDG (e.g. by adding a no. 2a). The authors propose to open the law regulating paid legal advice also to care and case managers (§ 10 para. 1 p. 1 no. 2a RDG), the necessary qualification requirements, personal suitability, liability insurance protection etc. and be implemented, analogous to § 12 RDG already applicable to other professional groups. Accordingly, the person must be of good repute, have liability insurance cover and prove theoretical - comprising at least 120 hours (§ 4 RDG) and - at least 2 years - knowledge for the sub-areas in which he or she intends to provide legal services.

## Data Availability

There is no data in the scientific sense that has any bearing on the background of the article, as it is a legal breakdown of the problem. It is therefore in the nature of things that there is already no data material that could be co-published. However, it is not necessary for the comprehensibility of the content.
