# Correction: Legal advice and care-effective use of care and case management: limits, risks and need for change

**DOI:** 10.1186/s12913-023-09097-0

**Published:** 2023-01-26

**Authors:** Thomas Ruppel, Max Georg Hügel, Simone Gloystein, Neeltje van den Berg

**Affiliations:** 1 University of Greifswald, Greifswald, Germany; 2 Bucerius Law School, Hamburg, Germany

**Correction: BMC Health Services Research 22, 1439 (2022)**

**https://doi.org/10.1186/s12913-022-08844-z**

Following publication of the original article [1], an error was identified in the article title: the funding note was erroneously included as part of the title. The funding note (funded by the innovation Fund of the Federal Joint Committee (G-BA) according to §§ 92a and 92b of the fifth book of the German social code (SGB V). Funding code 01NVF17029 (RubiN) regional uninterrupted in the network) was removed from the article title and the article title has been updated above and in the original article [1].
